# Specification curve analysis to identify heterogeneity in risk factors for dementia: findings from the UK Biobank

**DOI:** 10.1186/s12916-024-03424-w

**Published:** 2024-05-29

**Authors:** Renhao Luo, Dena Zeraatkar, Maria Glymour, Randall J. Ellis, Hossein Estiri, Chirag J. Patel

**Affiliations:** 1 Department of Biomedical Informatics, Harvard Medical School, Boston, MA USA; 2 Department of Anesthesia and Department of Health Research Methods, Evidence and Impact, McMaster University, Hamilton, Canada; 3 Department of Epidemiology, School of Public Health, Boston University, Boston, MA USA; 4 Department of Medicine, Harvard Medical School, Boston, MA USA

**Keywords:** Specification curve analysis, Dementia, All-cause dementia, Alzheimer’s disease, Vascular dementia, Frontotemporal dementia, Risk factors

## Abstract

**Background:**

In 2020, the *Lancet* Commission identified 12 risk factors as priorities for prevention of dementia, and other studies identified *APOE e4/e4* genotype and family history of Alzheimer’s disease strongly associated with dementia outcomes; however, it is unclear how robust these relationships are across dementia subtypes and analytic scenarios. Specification curve analysis (SCA) is a new tool to probe how plausible analytical scenarios influence outcomes.

**Methods:**

We evaluated the heterogeneity of odds ratios for 12 risk factors reported from the Lancet 2020 report and two additional strong associated non-modifiable factors (*APOE e4/e4* genotype and family history of Alzheimer’s disease) with dementia outcomes across 450,707 UK Biobank participants using SCA with 5357 specifications across dementia subtypes (outcomes) and analytic models (e.g., standard demographic covariates such as age or sex and/or 14 correlated risk factors).

**Results:**

SCA revealed variable dementia risks by subtype and age, with associations for TBI and *APOE e4/e4* robust to model specification; in contrast, diabetes showed fluctuating links with dementia subtypes. We found that unattributed dementia participants had similar risk factor profiles to participants with defined subtypes.

**Conclusions:**

We observed heterogeneity in the risk of dementia, and estimates of risk were influenced by the inclusion of a combination of other modifiable risk factors; non-modifiable demographic factors had a minimal role in analytic heterogeneity. Future studies should report multiple plausible analytic scenarios to test the robustness of their association. Considering these combinations of risk factors could be advantageous for the clinical development and evaluation of novel screening models for different types of dementia.

**Supplementary Information:**

The online version contains supplementary material available at 10.1186/s12916-024-03424-w.

## Background

In 2020, the *Lancet* Commission Report highlighted 12 potentially correlated and modifiable risk factors as targets for potential dementia prevention, intervention, and care for different age groups: education, hearing loss, traumatic brain injury (TBI), hypertension, alcohol consumption, obesity, smoking, depression, social isolation, physical inactivity, diabetes, and air pollution [1]. Additionally, previous studies identified non-modifiable risk factors, *APOE e4/e4* genotype [2], and family history of Alzheimer’s disease [3, 4], which are also strongly associated with dementia outcomes. While these factors can be prioritized based on the size of the risk (e.g., the magnitude of the odds ratio), it is unclear whether risk estimates are heterogeneous across demographics (e.g., age, gender, and ethnicity), operationalizations of dementia in the health record (e.g., as coded or non-coded subtypes, such as Alzheimer’s disease, and/or age of onset), or the co-occurrence/correlation of the major risk factors (e.g., a participant having both hypertension and diabetes).

Sources of heterogeneity in estimated effects of risk factors may be biological differences or the study design itself, such as the covariates selected as adjustment factors. Furthermore, given that the diagnosis and treatment of dementia involve an evolving, complex, and interdisciplinary approach in the clinical world and nonuniform progress among various populations [5], understanding the heterogeneity of dementia can offer a more robust basis for the early diagnosis and care of patients. In fact, the *Lancet* 2020 report has reported differences in risk across studies and demographic stratum. For example, the report documents large differences between study heterogeneity for traumatic brain injury (TBI) (*I* ^2^ of 99%) [1]. Digging in deeper, two studies on military veterans showed men have an increased dementia risk after TBI than women [6, 7]. Moreover, a Swedish study on TBI, adjusted for age, civil status, education, and pension, showed a larger risk of dementia than a Danish study, which only adjusted for sex [8, 9]. These results led to specific clinical care suggestions about TBI in the *Lancet* report, among other suggestions for other risk factors [1]. It is unclear what factors *within* studies contribute to the differences in risk estimates that emerge. Further still, the risk factors (e.g., obesity and diabetes) may be correlated with one another and provide “redundant” information. Modeling them together is required to attain an accurate risk estimate for one risk factor that is independent of the others.

Importantly, although there is unambiguous *theoretical* guidance about selecting covariates to estimate causal effects [10], applied researchers face tremendous ambiguity. Imperfect covariate measurement, unclear life course timing of occurrence of potential covariates compared to the exposure [11], and confusion of criteria for confounders versus mediators lead to inconsistent covariate sets across analyses. We claim that associations that are minimally influenced by the selection of alternative and plausible covariate sets are the most convincing. Moreover, the sensitivity and combinational effects of covariate sets are important to understand for future analyses [12]. Therefore, a *specification curve analysis* (SCA), an approach to analyze and visualize comprehensive sources of heterogeneity transparently, can enable us to identify specifications that are biological and clinically meaningful [13].

Here, we first apply SCA to systematically investigate the impact of 2 age groups, 5 different demographic variables, and 14 risk factors with a total of 1445 analytical specifications on the associations between risk factors and dementia-specific ICD 9/10 diagnosis codes [13, 14]. We considered specifications such as age groups and different dementia disease coding “subtypes” (as characterized by administrative International Classification of Disease [ICD] codes, pre-defined ICD codes for hospital admission records and death certificate records, and self-reported information), including AD, dementia that is unattributable to AD, frontotemporal dementia, or vascular dementia. For each, we compare models controlling for different covariate sets, such as adjustment for gender and/or ethnicity. Second, we selected risk factors across the spectrum of analytic robustness to test their association in multivariate modeling scenarios or different combinations of the risk factors themselves with a total of 3912 analytical specifications. Lastly, we examined the relationship between unattributed dementia patients and known dementia-type patients based on their risk factor profiles.

## Methods

### Study population

UK Biobank (UKB) is a detailed prospective study of 502,505 participants. The participants’ phenotypic and genetic information was collected between 2006 and 2010 when they were aged between 40 and 69 years in one of 22 assessment centers across England, Scotland, and Wales. During the visit, physical measurements were taken, and phenotypic information was collected by answering many questions about their health status and lifestyles via touch-screen or nurse-led questionnaires [15]. In addition, the participants’ genetic information was obtained from their biosamples, and the samples underwent genome-wide genotyping using the UK Biobank Axiom Array. This array directly measures approximately 850,000 variants, and more than 90 million variants were imputed using the Haplotype Reference Consortium and UK10K + 1000 Genomes reference panels [16]. All participants consent to the study. The UKB study application of this study is 52887. The Harvard internal review board (IRB) deemed the research to be non-human subjects research (IRB: IRB16-2145).

In this study, we only included participants greater than 45 years old in the analyses, which left 450,707 participants. We divided the study population into two different groups: midlife (age at dementia diagnosis between 45 and 65) and late-life (age at dementia diagnosis greater than 65), consistent with the 2020 *Lancet* report [1]. Additional file 2: Table S1 shows the definition used for each risk factor, and Additional file 2: Table S2 shows the dataset’s prevalence of participants with different types of dementia. The rest of the participant’s ages were defined as when they attended the first visit when grouping.

### Dementia case ascertainment

We focus on different dementia subtypes, including all-cause dementia, Alzheimer’s disease, vascular dementia, and frontotemporal dementia. The source of the report and the date of the report for all four types of dementia reports were obtained from the algorithmically defined dementia outcomes, which combine participants’ self-reported medical conditions, linked hospital diagnoses, and death registries provided by the UK Biobank group and validated by a different study [17, 18]. The patient’s diagnosis age is determined by the date of the specific dementia report date. The distribution of age at dementia diagnosis is shown in Additional file 1: Figure S1. For each subtype of dementia, the binary variable was defined as one if a given patient has that specific dementia diagnosis and zero if the patient does not have particular dementia subtypes. Additionally, we extracted “unattributed dementia” participants as the dementia participants who were in the all-cause dementia group but did not receive any specific diagnosis (e.g., ICD codes, hospital admission records, self-reports) for frontotemporal dementia, AD, and/or vascular dementia. The number of unattributed dementia is 1189 participants.

### Risk factors ascertainment

We identified the 31 *Lancet* Commission risk factor variables from 14 risk factors measured in the UK Biobank participants. We adopted the definitions of the modifiable variables from the 2020 *Lancet* report with some adjudication when extracting the data from the UK Biobank (see definitions in Additional file 2: Table S1). All risk factor variables were obtained from either the self-report or self-report combined with ICD 9/10 code diagnosis and other well-known clinical criteria when available (see prevalence of each risk factor in Additional file 2: Table S2). We used the risk factors with only self-reports available at the participants’ visits between 2006 and 2010, as shown in the questionnaires. For the risk factors with sources from self-reported and ICD code, we convert them into a binary table, and “Yes” annotates the participant as affirmative to the condition based on either source, whereas “No” annotates negative of the condition in the participant.

The *Lancet* Commission reported “social isolation” as one of the risk factors, and here, we use self-reported “loneliness” as a proxy of social isolation. We obtained the *APOE e4/e4* genotype information from the imputed chromosome 19 file provided by the UK Biobank and extracted the genotype for the two *APOE* SNPs, rs429358 and rs7412, by using PLINK2 (v2.00a3.1LM) [19]. Based on the previous literature, the *e4/e4* genotype corresponds to SNP alleles “CC” in rs429358 and “CC” in rs7412 [2]. After extracting the genotype information, we constructed a binary table for all participants, with “Yes” for carrying the allele and “No” for not carrying the allele.

### Specification curve analysis (SCA)

**Fig. 1:**
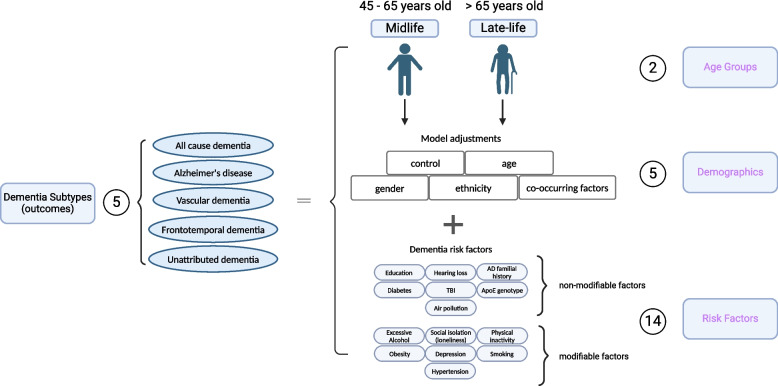
Specifications of risk in dementia subtypes. In our specification curve analysis (SCA), we modeled the dementia outcomes with three categories of experimental variables shown in purple, including “Age Groups,” “Demographics,” and “Risk Factors.” Specifically, we compared five different dementia subtypes, two age groups, five different model adjustments, and 14 different risk factors (31 different risk factor levels). Additionally, we selected robust risk factors and built models with different combinations of the risk factors for the subsequent SCA analysis. Figure created with Biorender

**Fig. 2:**
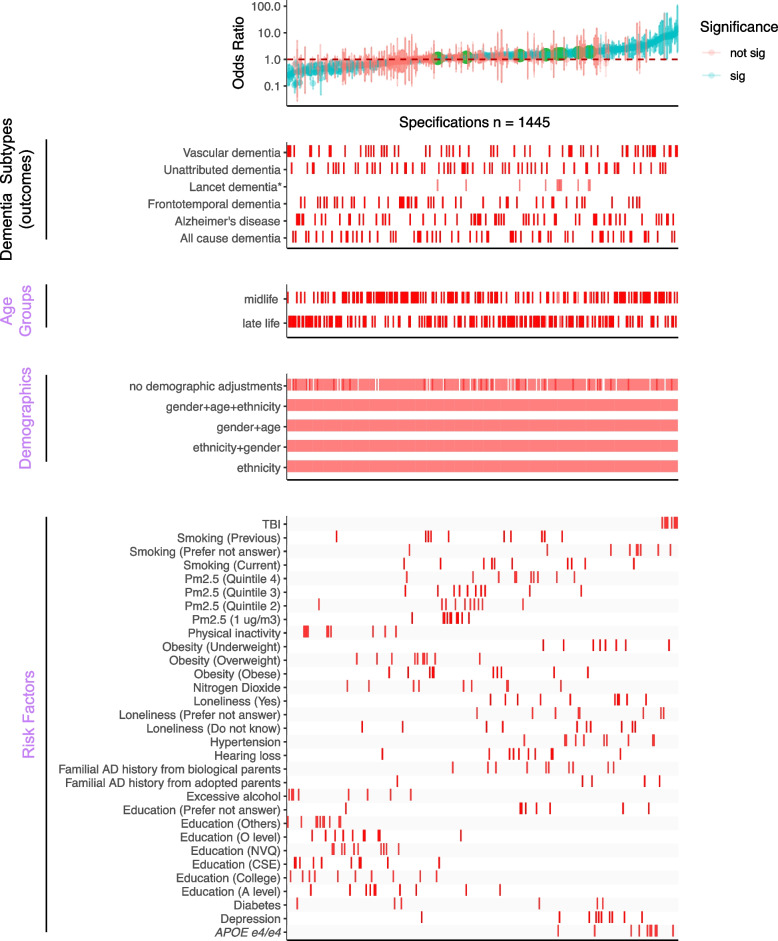
Overview of the SCA results for modeling dementia subtype outcomes with three categories of experimental variables, “Age Groups,” “Demographics,” and “Risk Factors.” All odds ratios are shown with 95% confidence intervals and colored by the significance of the model outputs in the top panel. The bottom four panels show the distribution of correspondents in each model. There are 1445 specifications from this SCA. “Sig” denotes nominal significance (*p* < 0.05). *Lancet dementia refers to the odds ratios reported from the 2020 *Lancet* report [1] shown in green circles in the top panel and is not included in the total specification number

**Fig. 3:**
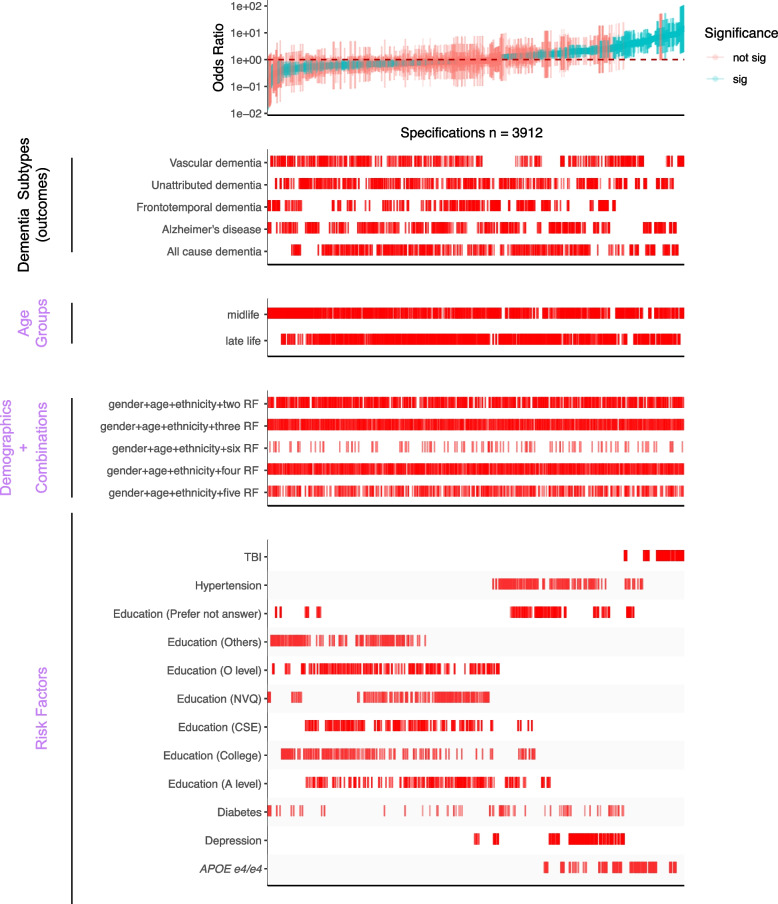
SCA results for modeling dementia subtype outcomes with three categories of experimental variables, including different combinations of selected risk factors (bottom panel). All odds ratios are shown with 95% confidence intervals and colored by the significance of the model outputs. There are a total of 3912 specifications from this SCA. “Sig” denotes nominal significance (*p* < 0.05). “RF” in the “Demographic + Combination” panel refers to selected risk factors in the “Risk Factors” panel

In this study, we conducted a “specification curve analysis”, which is an approach that systematically considers all reasonable analytical choices to address a particular research question [13]. “Specifications” may include, but are not limited to, covariate choice or causal model, inclusion criteria, definitions of the outcome (here, dementia subtypes), and the ways the risk factors are processed and cleaned. Here, we produced 5357 unique specifications, and these include all combinations of dementia subtype outcomes with three categories of experimental variables, specifically, 5 dementia subtypes, 2 age groups, 5 covariate or model choices, and 14 risk factors (31 risk factor variables) with/without combinations (Fig. 1). The risk factors were selected based on modifiable and non-modifiable factors suggested by the 2020 *Lancet* report [1], and non-modifiable genetic factors, *APOE e4/e4* [20], and family history of Alzheimer’s disease [3] that showed robust association with dementia. While adjusting for different demographics, we excluded 105 specifications, resulting in large confidence intervals, and have 1445 specifications for all risk factors. Additionally, we want to explore the combinatorial effects of different risk factors to mimic participants with multiple conditions. To do that, we selected risk factors with robust association (see the definition in the section below) and built models with multiple risk factors as the covariates and adjusted for gender, age, and ethnicity, resulting in an additional 3912 specifications. These specifications constituted our analysis set. We visualized the odds ratio (OR) results from each specification on a specification curve, providing a range of possible outcomes and allowing us to view the impact of our analytical choices on the results. The results of the specification curve analysis are shown in Fig. 2 for all risk factors (1445 specifications) and Fig. 3 with selected risk factors with combinational effects (3912 specifications). The odds ratios were the outcomes from different logistic regression models described in the section below.

### Threshold for assessment of “robust” associations

We applied a set of rules to claim a risk factor as “robust”: simply, given an outcome (e.g., all-cause dementia or AD), we deemed a risk factor as robust if the IQR of the OR is all on one side of no association (OR = 1). We only considered the risk factors that resulted in significant ORs. The selected risk factors were included in the combinational effect analysis, shown in Fig. 3.

### Statistical analyses

The study overview is shown in Fig. 1. All the analyses were performed using R v4.0.1 on the Harvard Medical School high-performance cluster. The prevalence of each risk factor was calculated by the number of positive cases of the risk factor, regardless of dementia status, divided by the total number of participants with the positive and negative of that risk factor. In this study, we built different logistic regression models corresponding to each dementia subtype in each age group, as shown in the results of SCA (Figs. 2 and 3). The gender and ethnicity information was extracted from the UKB data for all participants. We reported the odds ratio (OR), *p*-values, and 95% confidence interval based on the estimates from each model. We eliminated the risk factors for each age group with less than ten responses from the analyses. A risk factor is considered significant when the *p*-value is less than 0.05 (no adjustment for multiple hypotheses).

We excluded risk factors with less than 10 responses within the dementia category, and those are mainly “physical inactivity” and “excessive alcohol consumption” in midlife AD, vascular and frontotemporal dementia, and late-life in frontotemporal dementia. The summarized ORs for specifications are shown in Tables 1 and 2. The summary statistics for selected robust risk factors are shown in Additional file 2: Table S3. The age- and gender-adjusted ORs for each dementia subtype are shown in Additional file 2: Table S4. To test the statistical significance between ORs from the UK Biobank cohort and the *Lancet* report, we used the following formula to calculate *z* and obtained the *p*-value, *Z* = (beta_1 − beta_2)/sqrt(se(beta_1)^2^ + se(beta_2)^2^). Lastly, to examine the similarity of the ORs between different outcomes, we estimated Pearson’s correlation on the ORs between the unattributed dementia participants and the subtype participants and reported the correlation coefficients with the *p*-values.

**Table 1:** Median ORs for all SCA results with IQR

Risk factor	Age group	Median ORs	Quantile 25 ORs	Quantile 75 ORs	Significant (%)
APOE e4/e4	Late-life	4.18	3.94	5.83	100
APOE e4/e4	Midlife	3.05	1.99	3.85	60
Depression	Late-life	2.09	1.89	2.25	100
Depression	Midlife	2.11	2.04	2.33	80
Excessive alcohol	Late-life	0.32	0.29	0.38	80
Excessive alcohol	Midlife	0.73	0.57	0.86	20
Familial AD history from adopted parents	Late-life	1.88	0.82	1.93	0
Familial AD history from adopted parents	Midlife	4.61	3.20	6.02	50
Familial AD history from biological parents	Late-life	1.59	1.53	1.90	80
Familial AD history from biological parents	Midlife	1.33	1.20	1.47	40
Diabetes	Late-life	1.86	1.23	2.51	13
Diabetes	Midlife	0.52	0.37	0.96	0
Hypertension	Late-life	2.37	1.45	3.12	92
Hypertension	Midlife	1.69	1.61	2.18	80
Hearing loss	Late-life	1.49	1.05	1.57	56
Hearing loss	Midlife	1.48	1.42	1.56	60
Education (A level)	Late-life	0.55	0.46	0.84	48
Education (A level)	Midlife	0.81	0.65	1.05	0
Education (College)	Late-life	0.39	0.30	0.67	80
Education (College)	Midlife	0.63	0.58	0.80	40
Education (CSE)	Late-life	0.19	0.13	0.71	68
Education (CSE)	Midlife	0.60	0.59	0.75	32
Education (NVQ)	Late-life	0.40	0.34	0.80	68
Education (NVQ)	Midlife	0.69	0.69	0.74	24
Education (O level)	Late-life	0.48	0.42	0.60	84
Education (O level)	Midlife	0.62	0.60	0.70	40
Education (Others)	Late-life	0.40	0.33	0.62	92
Education (Others)	Midlife	0.45	0.33	0.46	80
Education (prefer not answer)	Late-life	1.40	1.31	1.53	45
Education (prefer not answer)	Midlife	1.93	1.16	2.85	25
Loneliness (do not know)	Late-life	1.12	0.79	1.71	20
Loneliness (do not know)	Midlife	2.47	1.97	2.88	40
Loneliness (prefer not to answer)	Late-life	1.63	1.51	1.82	0
Loneliness (prefer not to answer)	Midlife	4.98	2.66	6.89	60
Loneliness (yes)	Late-life	1.43	1.24	1.57	68
Loneliness (yes)	Midlife	2.46	2.40	2.68	100
Nitrogen dioxide	Late-life	1.13	0.89	1.33	0
Nitrogen dioxide	Midlife	1.08	0.85	1.26	0
Obesity (obese)	Late-life	1.05	0.95	1.15	40
Obesity (obese)	Midlife	1.29	1.26	1.33	36
Obesity (overweight)	Late-life	0.91	0.85	1.06	16
Obesity (overweight)	Midlife	0.93	0.63	1.03	0
Obesity (underweight)	Late-life	2.18	1.83	2.43	55
Obesity (underweight)	Midlife	2.10	1.87	3.47	25
Physical inactivity	Late-life	0.42	0.38	0.45	80
Physical inactivity	Midlife	0.64	0.46	0.73	20
Pm2.5 (1 µg/m^3^)	Late-life	1.10	1.09	1.15	80
Pm2.5 (1 µg/m^3^)	Midlife	1.16	1.13	1.17	40
Pm2.5 (quintile 2)	Late-life	1.21	1.19	1.23	28
Pm2.5 (quintile 2)	Midlife	1.10	1.08	1.16	0
Pm2.5 (quintile 3)	Late-life	1.27	1.19	1.47	48
Pm2.5 (quintile 3)	Midlife	1.10	1.06	1.20	0
Pm2.5 (quintile 4)	Late-life	1.39	1.29	1.49	68
Pm2.5 (quintile 4)	Midlife	1.43	1.15	1.45	40
Smoking (current)	Late-life	1.20	0.99	1.45	44
Smoking (current)	Midlife	1.66	1.34	1.89	60
Smoking (prefer not to answer)	Late-life	2.27	2.04	3.58	52
Smoking (prefer not to answer)	Midlife	4.21	2.15	6.37	75
Smoking (previous)	Late-life	1.49	1.17	1.63	72
Smoking (previous)	Midlife	1.01	0.97	1.04	0
TBI	Late-life	8.29	7.47	9.27	100
TBI	Midlife	8.43	7.19	10.58	100

**Table 2:** Median ORs for multivariate combinatios of risk factors SCA results and their IQR

Risk factor	Age group	Model group	Median ORs	Quantile 25 ORs	Quantile 75 ORs	Significant (%)
APOE e4/e4	Late-life	gender + age + ethnicity + five RF	4.82	4.25	6.08	84
APOE e4/e4	Late-life	gender + age + ethnicity + four RF	4.79	4.22	6.06	88
APOE e4/e4	Late-life	gender + age + ethnicity + six RF	4.82	4.57	6.07	80
APOE e4/e4	Late-life	gender + age + ethnicity + three RF	4.54	4.16	6.00	92
APOE e4/e4	Late-life	gender + age + ethnicity + two RF	4.27	4.14	5.92	96
APOE e4/e4	Midlife	gender + age + ethnicity + five RF	2.95	2.39	4.21	60
APOE e4/e4	Midlife	gender + age + ethnicity + four RF	2.95	2.06	4.20	60
APOE e4/e4	Midlife	gender + age + ethnicity + six RF	2.96	2.43	4.22	60
APOE e4/e4	Midlife	gender + age + ethnicity + three RF	3.06	2.03	4.18	60
APOE e4/e4	Midlife	gender + age + ethnicity + two RF	3.06	2.02	3.88	60
Depression	Late-life	gender + age + ethnicity + five RF	2.07	2.03	2.40	100
Depression	Late-life	gender + age + ethnicity + four RF	2.09	2.03	2.46	100
Depression	Late-life	gender + age + ethnicity + six RF	2.06	2.03	2.40	100
Depression	Late-life	gender + age + ethnicity + three RF	2.11	2.04	2.48	100
Depression	Late-life	gender + age + ethnicity + two RF	2.12	2.07	2.49	100
Depression	Midlife	gender + age + ethnicity + five RF	2.04	1.98	2.24	80
Depression	Midlife	gender + age + ethnicity + four RF	2.06	1.99	2.30	80
Depression	Midlife	gender + age + ethnicity + six RF	2.03	1.97	2.22	80
Depression	Midlife	gender + age + ethnicity + three RF	2.07	2.02	2.31	80
Depression	Midlife	gender + age + ethnicity + two RF	2.10	2.04	2.32	80
Diabetes	Late-life	gender + age + ethnicity + five RF	1.21	0.58	1.25	0
Diabetes	Late-life	gender + age + ethnicity + four RF	1.27	0.84	1.87	5
Diabetes	Late-life	gender + age + ethnicity + six RF	0.82	0.64	1.01	0
Diabetes	Late-life	gender + age + ethnicity + three RF	1.76	1.27	2.48	4
Diabetes	Late-life	gender + age + ethnicity + two RF	1.87	1.39	2.34	8
Diabetes	Midlife	gender + age + ethnicity + five RF	0.41	0.26	0.56	50
Diabetes	Midlife	gender + age + ethnicity + four RF	0.71	0.15	0.79	22
Diabetes	Midlife	gender + age + ethnicity + three RF	0.78	0.20	0.89	7
Diabetes	Midlife	gender + age + ethnicity + two RF	0.83	0.33	0.96	0
Hypertension	Late-life	gender + age + ethnicity + five RF	1.36	1.28	1.43	72
Hypertension	Late-life	gender + age + ethnicity + four RF	1.32	1.26	1.43	74
Hypertension	Late-life	gender + age + ethnicity + six RF	1.39	1.28	1.41	60
Hypertension	Late-life	gender + age + ethnicity + three RF	1.31	1.25	1.45	76
Hypertension	Late-life	gender + age + ethnicity + two RF	1.31	1.26	1.46	80
Hypertension	Midlife	gender + age + ethnicity + five RF	1.73	1.45	2.05	72
Hypertension	Midlife	gender + age + ethnicity + four RF	1.75	1.50	2.07	74
Hypertension	Midlife	gender + age + ethnicity + six RF	1.69	1.41	2.02	80
Hypertension	Midlife	gender + age + ethnicity + three RF	1.78	1.55	2.14	78
Hypertension	Midlife	gender + age + ethnicity + two RF	1.80	1.56	2.18	80
Education (A level)	Late-life	gender + age + ethnicity + five RF	0.77	0.63	0.82	0
Education (A level)	Late-life	gender + age + ethnicity + four RF	0.78	0.62	0.82	0
Education (A level)	Late-life	gender + age + ethnicity + six RF	0.75	0.64	0.80	0
Education (A level)	Late-life	gender + age + ethnicity + three RF	0.80	0.62	0.85	2
Education (A level)	Late-life	gender + age + ethnicity + two RF	0.80	0.75	0.85	4
Education (A level)	Midlife	gender + age + ethnicity + five RF	0.88	0.84	0.98	0
Education (A level)	Midlife	gender + age + ethnicity + four RF	0.88	0.78	0.95	0
Education (A level)	Midlife	gender + age + ethnicity + six RF	0.92	0.86	1.08	0
Education (A level)	Midlife	gender + age + ethnicity + three RF	0.84	0.72	0.90	0
Education (A level)	Midlife	gender + age + ethnicity + two RF	0.84	0.67	0.88	0
Education (College)	Late-life	gender + age + ethnicity + five RF	0.62	0.61	0.65	80
Education (College)	Late-life	gender + age + ethnicity + four RF	0.62	0.55	0.70	80
Education (College)	Late-life	gender + age + ethnicity + six RF	0.63	0.62	0.65	80
Education (College)	Late-life	gender + age + ethnicity + three RF	0.61	0.54	0.70	80
Education (College)	Late-life	gender + age + ethnicity + two RF	0.59	0.55	0.69	80
Education (College)	Midlife	gender + age + ethnicity + five RF	0.67	0.53	0.78	36
Education (College)	Midlife	gender + age + ethnicity + four RF	0.65	0.53	0.78	40
Education (College)	Midlife	gender + age + ethnicity + six RF	0.68	0.54	0.80	20
Education (College)	Midlife	gender + age + ethnicity + three RF	0.64	0.53	0.76	40
Education (College)	Midlife	gender + age + ethnicity + two RF	0.63	0.53	0.76	40
Education (CSE)	Late-life	gender + age + ethnicity + five RF	0.78	0.73	0.88	4
Education (CSE)	Late-life	gender + age + ethnicity + four RF	0.78	0.70	0.86	8
Education (CSE)	Late-life	gender + age + ethnicity + six RF	0.79	0.78	0.90	0
Education (CSE)	Late-life	gender + age + ethnicity + three RF	0.74	0.69	0.83	12
Education (CSE)	Late-life	gender + age + ethnicity + two RF	0.73	0.69	0.82	16
Education (CSE)	Midlife	gender + age + ethnicity + five RF	0.63	0.61	0.80	8
Education (CSE)	Midlife	gender + age + ethnicity + four RF	0.63	0.60	0.79	14
Education (CSE)	Midlife	gender + age + ethnicity + six RF	0.63	0.61	0.80	0
Education (CSE)	Midlife	gender + age + ethnicity + three RF	0.62	0.59	0.78	20
Education (CSE)	Midlife	gender + age + ethnicity + two RF	0.62	0.60	0.76	20
Education (NVQ)	Late-life	gender + age + ethnicity + five RF	0.87	0.84	0.92	4
Education (NVQ)	Late-life	gender + age + ethnicity + four RF	0.85	0.82	0.88	8
Education (NVQ)	Late-life	gender + age + ethnicity + six RF	0.91	0.87	0.92	0
Education (NVQ)	Late-life	gender + age + ethnicity + three RF	0.83	0.82	0.86	12
Education (NVQ)	Late-life	gender + age + ethnicity + two RF	0.82	0.81	0.83	16
Education (NVQ)	Midlife	gender + age + ethnicity + five RF	0.77	0.67	0.85	4
Education (NVQ)	Midlife	gender + age + ethnicity + four RF	0.74	0.67	0.84	8
Education (NVQ)	Midlife	gender + age + ethnicity + six RF	0.79	0.69	0.88	0
Education (NVQ)	Midlife	gender + age + ethnicity + three RF	0.71	0.66	0.80	14
Education (NVQ)	Midlife	gender + age + ethnicity + two RF	0.70	0.67	0.75	20
Education (O level)	Late-life	gender + age + ethnicity + five RF	0.81	0.78	0.84	44
Education (O level)	Late-life	gender + age + ethnicity + four RF	0.79	0.63	0.82	48
Education (O level)	Late-life	gender + age + ethnicity + six RF	0.82	0.82	0.84	40
Education (O level)	Late-life	gender + age + ethnicity + three RF	0.77	0.61	0.81	52
Education (O level)	Late-life	gender + age + ethnicity + two RF	0.76	0.60	0.79	56
Education (O level)	Midlife	gender + age + ethnicity + five RF	0.68	0.57	0.73	24
Education (O level)	Midlife	gender + age + ethnicity + four RF	0.67	0.56	0.72	34
Education (O level)	Midlife	gender + age + ethnicity + six RF	0.69	0.57	0.74	0
Education (O level)	Midlife	gender + age + ethnicity + three RF	0.66	0.56	0.71	38
Education (O level)	Midlife	gender + age + ethnicity + two RF	0.65	0.61	0.69	40
Education (others)	Late-life	gender + age + ethnicity + five RF	0.73	0.69	0.75	76
Education (others)	Late-life	gender + age + ethnicity + four RF	0.72	0.68	0.73	80
Education (others)	Late-life	gender + age + ethnicity + six RF	0.75	0.74	0.75	60
Education (others)	Late-life	gender + age + ethnicity + three RF	0.70	0.66	0.72	80
Education (others)	Late-life	gender + age + ethnicity + two RF	0.68	0.64	0.70	80
Education (others)	Midlife	gender + age + ethnicity + five RF	0.44	0.33	0.49	76
Education (others)	Midlife	gender + age + ethnicity + four RF	0.44	0.33	0.49	78
Education (others)	Midlife	gender + age + ethnicity + six RF	0.45	0.35	0.49	80
Education (others)	Midlife	gender + age + ethnicity + three RF	0.45	0.33	0.48	80
Education (others)	Midlife	gender + age + ethnicity + two RF	0.45	0.33	0.47	80
Education (prefer not to answer)	Late-life	gender + age + ethnicity + five RF	1.60	1.51	1.66	60
Education (prefer not to answer)	Late-life	gender + age + ethnicity + four RF	1.56	1.50	1.65	58
Education (prefer not to answer)	Late-life	gender + age + ethnicity + six RF	1.63	1.56	1.66	50
Education (prefer not to answer)	Late-life	gender + age + ethnicity + three RF	1.54	1.51	1.61	60
Education (prefer not to answer)	Late-life	gender + age + ethnicity + two RF	1.54	1.51	1.59	65
Education (prefer not to answer)	Midlife	gender + age + ethnicity + five RF	1.85	1.12	2.79	25
Education (prefer not to answer)	Midlife	gender + age + ethnicity + four RF	1.84	1.12	2.83	25
Education (prefer not to answer)	Midlife	gender + age + ethnicity + six RF	1.85	1.08	2.71	25
Education (prefer not to answer)	Midlife	gender + age + ethnicity + three RF	1.84	1.12	2.86	25
Education (prefer not to answer)	Midlife	gender + age + ethnicity + two RF	1.82	1.13	2.84	25
TBI	Late-life	gender + age + ethnicity + five RF	5.12	4.78	7.94	100
TBI	Late-life	gender + age + ethnicity + four RF	6.90	4.84	7.96	100
TBI	Late-life	gender + age + ethnicity + six RF	5.00	4.56	5.95	100
TBI	Late-life	gender + age + ethnicity + three RF	7.18	4.97	7.92	100
TBI	Late-life	gender + age + ethnicity + two RF	7.23	6.76	7.94	100
TBI	Midlife	gender + age + ethnicity + five RF	8.64	8.29	13.06	94
TBI	Midlife	gender + age + ethnicity + four RF	8.60	7.70	12.63	88
TBI	Midlife	gender + age + ethnicity + six RF	8.54	8.44	11.11	100
TBI	Midlife	gender + age + ethnicity + three RF	8.53	7.26	12.34	89
TBI	Midlife	gender + age + ethnicity + two RF	8.47	7.09	11.61	84

## Results

Through two specification curve analyses—one encompassing a comprehensive range of established dementia risk factors and the other focusing on a subset of particularly stable risk factors to study combinational effects—we identified heterogeneity in the risk for various dementia subtypes and across different age groups. Nonetheless, modifications in age categorization or demographic factors exerted negligible influence on the associations between risk determinants and dementia manifestations. Of particular significance were traumatic brain injury (TBI) and the *APOE e4/e4* allele, which demonstrated consistent associations with all examined dementia subtypes across all analytical conditions. In contrast, risk factors such as diabetes exhibited variable correlations with diverse dementia outcomes. Furthermore, individuals with unattributed dementia participants displayed risk profiles that were analogous to those with definitive subtypes, transcending age delineations.

In this study, we included 450,707 participants from the UK Biobank with ages greater than 45 years old and divided them into two groups: midlife (45–65 years old) and late-life (greater than 65 years old). The midlife group consists of about 83% of all the participants. UK Biobank participants accrued 2710 all-cause dementia (44.5% female, about 0.6% of all UK Biobank participants), 1005 Alzheimer’s Disease (48.9% female), 539 vascular dementia (36.4% female), and 113 frontotemporal dementia (43.4% female) diagnoses during follow-up. There are 20%, 13%, 26.5%, and 12.4% of all-cause dementia, Alzheimer’s disease, frontotemporal dementia, and vascular dementia participants in the midlife group. The results from SCA analyses are summarized by the median ORs, their interquartile ranges (IQR), and the percentage of significant model outputs in Tables 1 and 2. The definitions we used to query the risk factors are shown in Additional file 2: Table S1. Details about the number of participants included for each risk factor among different age groups and prevalence can be found in Additional file 1: Figure S1 and Additional file 2: Table S2.

### Specification curve analysis to illustrate analytic heterogeneity in different models

Our comprehensive specification curve analysis revealed significant findings regarding dementia risk factors. The comprehensive specifications consist of three categories of experimental variables to model the dementia subtypes as the outcome, outlined in Fig. 1. Out of 1445 models in Fig. 2, 716 showed statistically significant results (*p* < 0.05). Considering the directionality, the median OR of all the significant models greater than or equal to 1 is 2.09 [1.49–3.39], and less than 1 is 0.42 [0.33–0.58]. There are 917 models with ORs greater than or equal to 1.

We presented the details of the comprehensive specifications of our models in Table 1. The age groups showed that more midlife participants contributed to large ORs than late-life participants. We did not observe distinguishable differences in estimates depending on covariate sets in any specifications. For the same risk factor (e.g., diabetes) under different model adjustments, the odds ratio varied. Diabetes had inconsistent associations in different age groups (midlife median OR 0.52 [0.37–0.96] and late-life median OR 1.86 [1.22–2.51]). On the other hand, most other risk factors had similar associations with dementia regardless of the age group evaluated. PM2.5 ORs differed based on analytic specification; for example, the top quintile has a midlife median OR 1.43 [1.15–1.45] and a late-life median OR 1.39 [1.29–1.49], which is higher compared to the second quintile (midlife median OR 1.10 [1.08–1.16] and late-life median OR 1.21 [1.19–1.23]).

The *Lancet* study’s risk factors had varying levels of robustness, with some showing smaller odds ratios (ORs) in the UK Biobank (Additional file 1: Figure S2). Traumatic brain injury (TBI) showed a median OR of 8.43 [7.19–10.58] for midlife and 8.29 [7.47–9.27] for the late-life group in both studies, with significant midlife OR difference. Depression ORs were higher in UK Biobank across all ages (midlife = 2.11 [2.04–2.32], late-life = 2.09 [1.89–2.25]), while hypertension ORs were higher in the *Lancet* but lower in UK Biobank (midlife = 1.69 [1.61–2.18], late-life: 2.37 [1.45–3.12]). No significant late-life risk factor differences were noted. Inconsistencies in excessive alcohol consumption and physical inactivity associations between studies may relate to UK Biobank’s smaller sample size, as shown in Additional file 2: Table S2. These disparities suggest population differences as a potential cause of heterogeneity in risk factor impacts. Furthermore, we are interested in studying the impacts of multiple risk factors in one model on the ORs.

### Specification curve analyses highlight several robust risk factors in association with dementia outcomes

**Fig. 4:**
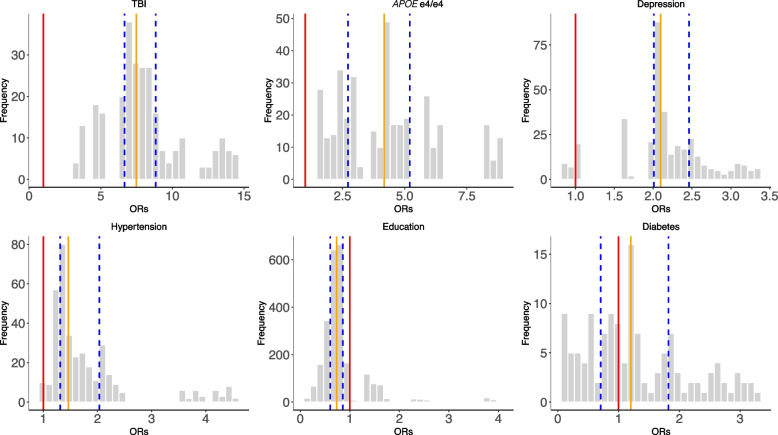
The distributions of odds ratios among the selected risk factors across all combinations of SCA analyses (Fig. 3). The red line represents OR = 1, the orange line represents the median ORs, and the blue lines define the IQRs

To test the robustness of six selected risk factors (TBI, *APOE e4/e4*, hypertension, diabetes, depression, and education) from the previous specification curve analyses, we executed 3912 multivariate logistic regression models with different combinations of the selected risk factors (while adjusting for age, gender, and ethnicity) (Fig. 3 and Table 2). In these multivariate models, we assessed each risk factor by (a) the number of times the OR is greater than or less than 1 and (b) the interquartile range of the OR (Fig. 4 and Additional file 2: Table S3) to assess their heterogeneity.

In Fig. 4, we showed the distribution of the ORs among the selected risk factors. 100% of models that included *APOE e4/e4* (ORs = 1.56–8.76), 100% of models that included TBI (ORs = 3.42–14.48), 97% of models with hypertension (ORs = 0.97–4.54), and 93% depression outputs (ORs = 0.87–3.36) are positively associated (OR > 1) with dementia outcomes. Further, higher than high school education categories were all negatively (OR < 1) associated with dementia outcomes (e.g., 84% in A level, 96% in O level, and 90% in college and CSEs). In contrast, diabetes has mixed and non-robust model outputs (ORs = 0.11–3.28; 55% of models have ORs less than 1) from the combinations of risk factors, suggesting inconsistent association trends for diabetes in disease outcomes in the presence of other correlated risk factors.

To assess the robustness of these risk factors (except diabetes) in different dementia subtypes, we compared the OR generated from the no-demographic-adjusted models with the odds ratios from the multivariate models with all six risk factors (Additional file 1: Figure S3). We observed positive correlations between the simple and multi-risk factor models in all dementia subtypes. The highest correlation was in unattributed dementia (*R* ^2^ = 0.995, *p*-value = 2.425e − 10), and frontotemporal dementia showed the lowest correlation (*R* ^2^ = 0.694, *p*-value = 0.002). These findings suggest the robustness of the five risk factors’ associations with dementia outcomes, regardless of model adjustments.

### Heterogeneity of risk factors within and across different age groups and dementia subtypes

**Fig. 5:**
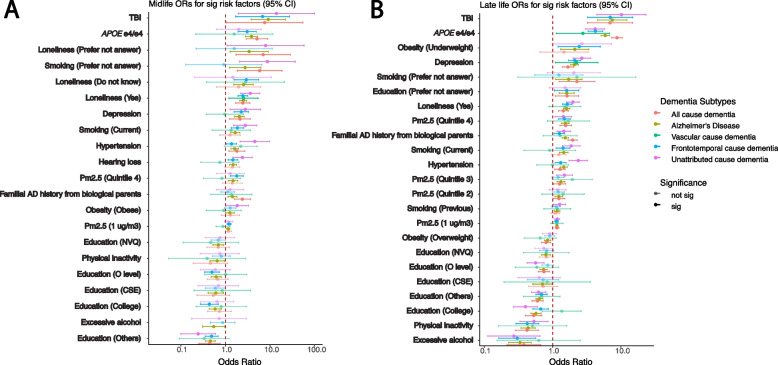
Heterogeneity between different dementia subtypes among significant risk factors identified in age- and gender-adjusted models from the all-cause dementia participants in respective age groups. The ORs and 95% confidence intervals from gender- and age-adjusted models from significant all-cause dementia outputs ranked by the ORs from high to low in midlife (**A**) and late-life (**B**) groups. The ORs in the plots are colored by the dementia subtypes, and transparency indicates the significance

Next, we compared the ORs for the risk factors within each age group with different justifications (Fig. 5, Additional file 1: Figure S4, and Additional file 2: Table S4). The range of ORs in the age- and gender-adjusted models for *APOE e4/e4* in midlife is from 1.57 to 5.09, and in late-life is from 2.77 to 8.78. Similarly, the ranges of ORs for TBI in age- and gender-adjusted models are 6.81 to 13.97 for midlife and 6.92 to 10.15 for late life. In contrast, some risk factors present small differences between dementia subtypes, such as continuous PM2.5 (1 µg/m3). The OR range in PM2.5 (per 1 µg/m3) is in the age- and gender-adjusted model in midlife which is between 0.88 and 1.21, and the range in late life is between 1.13 and 1.17.

There are risk factors that have a significant association (*p* < 0.05) in one age group but not in the other one, and vice versa. Hearing loss is significant in all-cause dementia in the midlife group but becomes non-significant in late life. Overweight is significant in the late-life group for all-cause dementia participants but not in the midlife group. Additionally, within subtypes, some risk factors differ across age groups. For example, in the age- and gender-adjusted model (Fig. 5), *APOE e4/e4* has significant ORs, from high to low, in AD (5.09 [2.92–8.87]), all-cause dementia (3.85 [2.82–5.24]), and unattributed cause dementia (3.08 [1.93–4.9]) among the midlife participants. Moreover, the *APOE e4/e4* ORs are significant among the late-life participants, displaying a different OR ranking and overall larger ORs compared to the midlife group in the age- and gender-adjusted models: AD (8.78 [7.29–10.57]), all-cause dementia (5.90 [5.13–6.78]), unattributed cause dementia (4.23 [3.31–5.40]), vascular dementia (4.19 [3.03–5.79]), and frontotemporal dementia (2.77 [1.12–6.86]). Similarly, intra-group variation in OR rankings also exists in other risk factors, including depression, diabetes, hypertension, loneliness, and TBI.

### Heterogeneity of risk factors for unattributed dementia

In the UKB, there are 1189 participants out of 2732 all-cause dementia participants who were not coded into the dementia subtypes (specify how you classified unattributed dementia). We refer to those participants as “unattributed” dementia participants. To understand the risk factors for those participants, we calculated the ORs for those people separately for each age group (Additional file 2: Table S4). Similar to other dementia subtypes, TBI (OR with 95% CI for midlife 6.82 [1.69–27.48] and for late-life OR 6.98 [3.25–14.97]) and *APOE e4/e4* genotypes (OR with 95% CI for midlife 3.08 [1.93–4.90] and for late-life OR 4.23 [3.31–5.40]) were among the top risk factors with high odds ratios. Moreover, the rankings of the risk factors in both midlife and late-life groups are similar to the all-cause dementia participants. In midlife, familial AD history from adopted parents, loneliness, depression, smoking, hearing loss, hypertension, and quintile 4 of the PM2.5 are the significant risk factors with ORs greater than 1. In late life, underweight, depression, loneliness, smoking, quintile 4 of the PM2.5, and familial AD history from biological parents have significant odds ratios in all models with ORs greater than 1. Significant education levels have ORs less than 1 in both age groups.

**Fig. 6:**
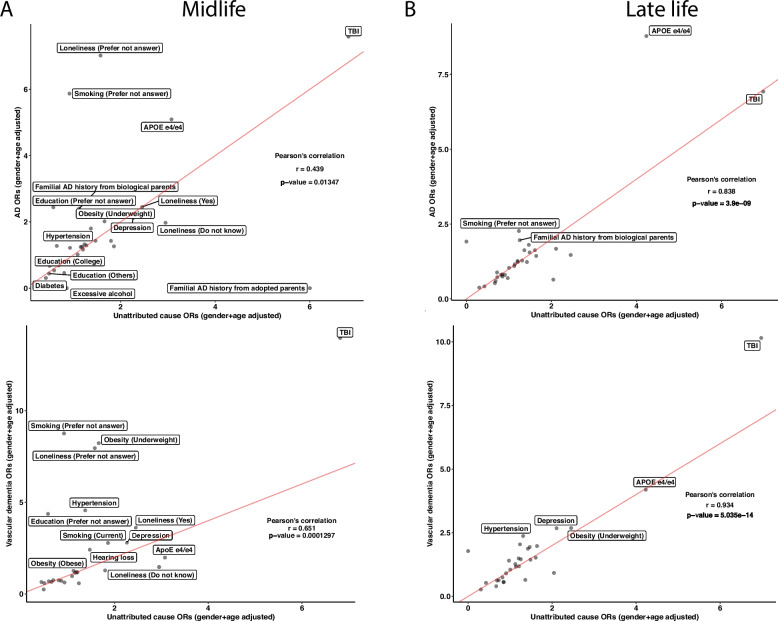
Unattributed cause dementia ORs in all risk factors and their correlations with known cause dementia. We subset the participants with unattributed causes of dementia and reported the ORs on the risk factors in both midlife and late life. We ran correlations on the ORs between the known subtypes of dementia with unattributed causes of dementia in midlife (**A**) and late-life (**B**) in AD and vascular dementia. Lastly, we calculated Pearson’s correlation for each comparison and reported the correlation coefficients and the *p*-values. The red lines in the correlation plots show when the slope is 1. There are *N* = 131, 67, and 296 participants in the midlife group for AD, vascular dementia, and unattributed cause dementia, respectively, and *N* = 874, 472, and 873 participants in the late-life group for AD, vascular dementia, and unattributed cause dementia, respectively

To further investigate the concordance of risk factors between the unattributed dementia participants and known dementia participants, we correlated the ORs of the risk factors between unattributed dementia to each other specific subtype. In the midlife group, the unattributed dementia group had Pearson correlation coefficients of 0.439, 0.651, and 0.703 with vascular dementia, AD, and frontotemporal dementia, respectively (Fig. 6A and Additional file 1: Figure S5A). While most risk factors have similar ORs in the comparisons, some risk factors, such as *APOE e4/e4*, showed the highest ORs across specifications. In the vascular dementia comparison, the *APOE e4/e4* has a higher OR in the unattributed dementia cause group than in vascular dementia. In contrast, it has lower ORs in unattributed dementia when compared to AD. The ORs from unattributed participants significantly correlated with those in AD and vascular dementia participants with Pearson’s correlation coefficients of 0.838 and 0.934, respectively (Fig. 6B and Additional file 1: Figure S5B), whereas a low correlation with the frontotemporal dementia participants with a correlation coefficient of 0.491. In all the late-life comparisons, both TBI and *APOE e4/e4* have high ORs. Based on the Pearson correlation results, the unattributed participants are more correlated with participants having frontotemporal dementia in the midlife group and vascular dementia and AD in the life group.

## Discussion

In this study, we attempt to disentangle the heterogeneity in dementia arising from variation in examining a number of phenotypes or subtypes and adjustment covariates using the tools of the “specification curve analysis” (SCA). Analytic decisions made during modeling risk factors associated with dementia are dependent on assumptions, and these assumptions may bias results and induce heterogeneity. We performed SCA to study how all articulated analytical specifications (e.g., classification of subtype, age of diagnosis, and risk factors in a multivariate model) may affect the OR estimates and their precision. The space of possible assumptions we explore includes both study designs, for example, (a) covariates and risk factors in the model; (b) outcomes, such as all-comers dementia and ICD-encoded Alzheimer’s disease; and (c) age at the outcome. In our study, we systematically examined 5357 specifications, including 1445 specifications for all risk factors and 3912 specifications for selected risk factors with combinations. These specifications and factors are reasonable assumptions to make when assessing evidence for dementia but are not made explicit when developing recommendations such as the *Lancet* 2020 report (we considered all specifications from the report in the present study). For instance, we included both known modifiable (such as smoking and physical inactivity) and non-modifiable risk factors (such as TBI and air pollution) for dementia in our specifications for modeling. The specification curves are important to document to understand sources of variation in OR estimates rather than one monolithic estimate. Specifically, our results highlighted risk factors that consistently have high odds ratios across dementia types but also showed the risk factors with highly varying ORs across covariates, age groups, and dementia outcomes. Several risk factors remained robust when modeled in complex multivariate scenarios (e.g., TBI and *APOE e4/e4*). This database of specifications may provide a map for future researchers to guide future study plans in their cohort studies to identify modifiable risk factors in dementia systematically.

### Examples of heterogeneity and robust associations

We discuss several specific associations between risk factors and dementia. TBI, we found, had a high OR across all specifications (median OR across risk factor specifications of midlife 8.29 [7.47–9.27] and late-life 8.43 [7.19–10.58]) and a large range across different combinations of specifications in the late-life group with median OR ranged from 5 (modeling 6 concurrent risk factors) to 7.23 (modeling 2 concurrent risk factors). In other studies, we also observe large OR ranges [6, 21, 22]. For instance, the *Lancet* 2020 reported an OR of only 1.8 [1.5–2.2] [1]. Moreover, the comparison between our results and the *Lancet* report also showed heterogeneity among different populations. The MIRAGE study, on the other hand, reported that participants (average age 69.9 ± 9) with head injury have an OR of 4.0 [2.9–5.5] [23]. In addition, the *APOE e4/e4* genotype is strongly associated with dementia risk in all specifications. The median ORs across all specifications are 3.05 [1.99–3.85] for the midlife group and 4.18 [3.84–5.83] for the late-life group. This strong association is in line with previous studies and well documented by those studies [24].

Previous studies on hypertension [25], diabetes [26, 27], and depression [28] have claimed causal correlations with dementia. In our study, diabetes has a variety of associations, where the ORs were larger than 1 and less than 1 depending on the analytic scenario, whereas hypertension and depression are examples of more robust and consistent associations with respect to the direction of OR. The degree of heterogeneity implies that these risk factors will require larger sample sizes and more precise outcome measures, potentially beyond ICD codes, to estimate their causal association, if any. These results also suggested that the large heterogeneity in diabetes (and other non-robust risk factors) could be potentially due to the different specifications and their correlation with other risk factors. Another group of robust associations is education. In our analysis, having a higher education level, including college and other professional training (e.g., nursing), mostly resulted in a negative association with dementia outcomes in all specifications. However, this relationship is not linear (e.g., the risks are different between A-level and CSE), which aligns with the previous results [29].

### The role of outcome coding on heterogeneity of odds ratios

The lack of robustness of risk estimates can be driven by clinical/biological differences, such as subtypes of dementia, including Alzheimer’s disease (AD), vascular dementia, and frontotemporal dementia. However, these differing outcomes are represented by a set of disease codes in a health registry setting despite their complex clinical manifestation. We emphasize it is impossible to know the true diagnosis or subtype of dementia in these cases.

To surmount the obstacles associated with dementia classification, one viable strategy may involve the refinement of diagnostic codes to encompass not only the clinical manifestations observable through current standards, neuroimaging, and biomarkers [5] but also data pertaining to risk factors. By adopting this approach, a more comprehensive profile of dementia could be documented, thereby enriching the foundation for future dementia research.

Relatedly, our method of incorporating various combinations of risk factors, such as TBI, *APOE e4/e4*, hypertension, etc., indicates that these robust association risk factors should be evaluated collectively to assess dementia risk and diagnosis accurately. Evaluating these factors individually may result in biased assessments or assessments that underperform in accuracy. Considering these risk factor combinations could be clinically beneficial in developing and appraising new models for screening for dementia subtypes.

In the analyzed instance of the UK Biobank, 1189 participants (43.5% of all-cause dementia participants) were not categorized into one of the known dementia subtypes by ICD codes. These participants have specification curves that resemble vascular dementia and AD participants [15].

### Implications

In this investigation, we have identified and quantified analytic sources of analytic heterogeneity in risk factors for AD. We hypothesize that the heterogeneity in ORs that arise from age and subtypes may be due to differing etiologies between subtypes or “errors” in subtyping, such as misclassification.

Secondly, risk estimates for individual factors often neglect adjustments that account for correlations with other risk factors. Given that modifiable risk factors considered in *Lancet* 2020 are interrelated [30, 31], it is crucial to analyze them concurrently in a multivariate regression model to obtain independent estimates. While the role of age is known in *APOE*-derived risk for AD [20], the role of age in modifiable risk factors is elusive. Future investigations should estimate the degree to which subtype heterogeneity is driven by analytic specification, such as inclusion criteria, versus biological differences. Specifically, future studies could validate the combinational effects in a different dataset, such as the All of Us cohort [32], to test if the robustness of risk factors could be replicated and generalizable. Additionally, incorporating the current diagnosis methods, neuroimaging, and biomarker testing into the specifications would also help to improve the accuracy of diagnosing cognitive decline and dementia not captured via the disease codes utilized in this study.

Thirdly, while we identified how risk factors might differ across subtypes, what remains is how intervening on any one or multiple factors might induce change in risk [33]. For example, a few studies have tried to establish the causal relationship between risk factors and dementia outcomes, but they resulted in either positive or negative associations when different assumptions were considered [34–36].

### Strength and limitations

Here, we compared the odds ratios of modifiable and non-modifiable risk factors directly in different age groups and studied the changes in different risk factors between age groups to highlight differences and similarities between the UK Biobank population and the *Lancet* report [1]. The SCA highlighted the different effects of each risk factor, age group, and subtype of dementia described by different models. Future surveillance programs should describe the odds ratios of these factors as the case mix changes. For example, in a similar study examining the *Lancet* reported dementia risk factors on the US population, different ethnicities yielded different population attributable fractions (PAF) [21]. As a result, comprehensive assessments of dementia patients using multiple techniques should be taken to classify and later validate subtypes of dementia.

In this study, the sample size for certain risk factors was small. Moreover, we used self-reported data to ascertain non-genomic risk factors (Additional file 2: Table S1), which may limit the replicability of our findings in other datasets due to variability in questionnaire design. Third, a previous study showed that the positive predicted values for all-cause dementia and dementia subtype cases vary [18], which may also impact the replicability of the results in different datasets and the general population.

## Conclusions

In the current study, we observed heterogeneity in the risk of dementia, and estimates of risk factors were influenced by the inclusion of a combination of other risk factors but not demographic factors. It is important for future recommendations and reports of risk to include multiple plausible analytical scenarios that consider correlated risk factors to assess the strength and accuracy of risk estimates.

### Supplementary Information

Additional file 1: Figure S1. Distribution of participants’ age used in this study. Figure S2. Comparison of the risk factors’ odds ratios between UKB and the Lancet 2020 report. Figure S3. Correlation between the simple and complex models for dementia subtypes. Figure S4. Non-significant risk factors results across different dementia subtypes, in complementary to Fig. 5. Figure S5. Correlation between frontotemporal dementia and the unattributed cause of dementia participants.

Additional file 2: Supplementary Table S1. Definitions of risk factors. Supplementary Table S2. Prevalence of different dementia risk factors in different age groups. Supplementary Table S3. Summary stats for odds ratios from selected risk factors (Fig. 4). Supplementary Table S4. Gender and age adjusted model outputs in midlife and late life group.

## Data Availability

All the results of this study are included in the figure or in the supplementary information. The UK Biobank dataset is not publicly available. Researchers interested in accessing the dataset must apply and be approved by the UK Biobank management team. See details: https://www.ukbiobank.ac.uk/enable-your-research/apply-for-access.

## Abbreviations

AD: Alzheimer’s disease
CI: Confidence interval
ICD: International Classification of Diseases
OR: Odds ratio
PAF: Population attributable fractions
SCA: Specification curve analysis
TBI: Traumatic brain injury
UKB: UK Biobank
