# Supplementary material for: Specification curve analysis to identify heterogeneity in risk factors for dementia: findings from the UK Biobank
Source: BMC Med. 2024 May 29;22:216. doi: 10.1186/s12916-024-03424-w (PMC11134914; doi:10.1186/s12916-024-03424-w)
Supplement: Supplementary file 1 — Additional file 1: Figure S1. Distribution of participants’ age used in this study. Figure S2. Comparison of the risk factors’ odds ratios between UKB and the Lancet 2020 report. Figure S3. Correlation between the simple and complex models for dementia subtypes. Figure S4. Non-significant risk factors results across different dementia subtypes, in complementary to Fig. 5. Figure S5. Correlation between frontotemporal dementia and the unattributed cause of dementia participants. [file 12916_2024_3424_MOESM1_ESM.docx]

Additional File 1: Supplementary Figures


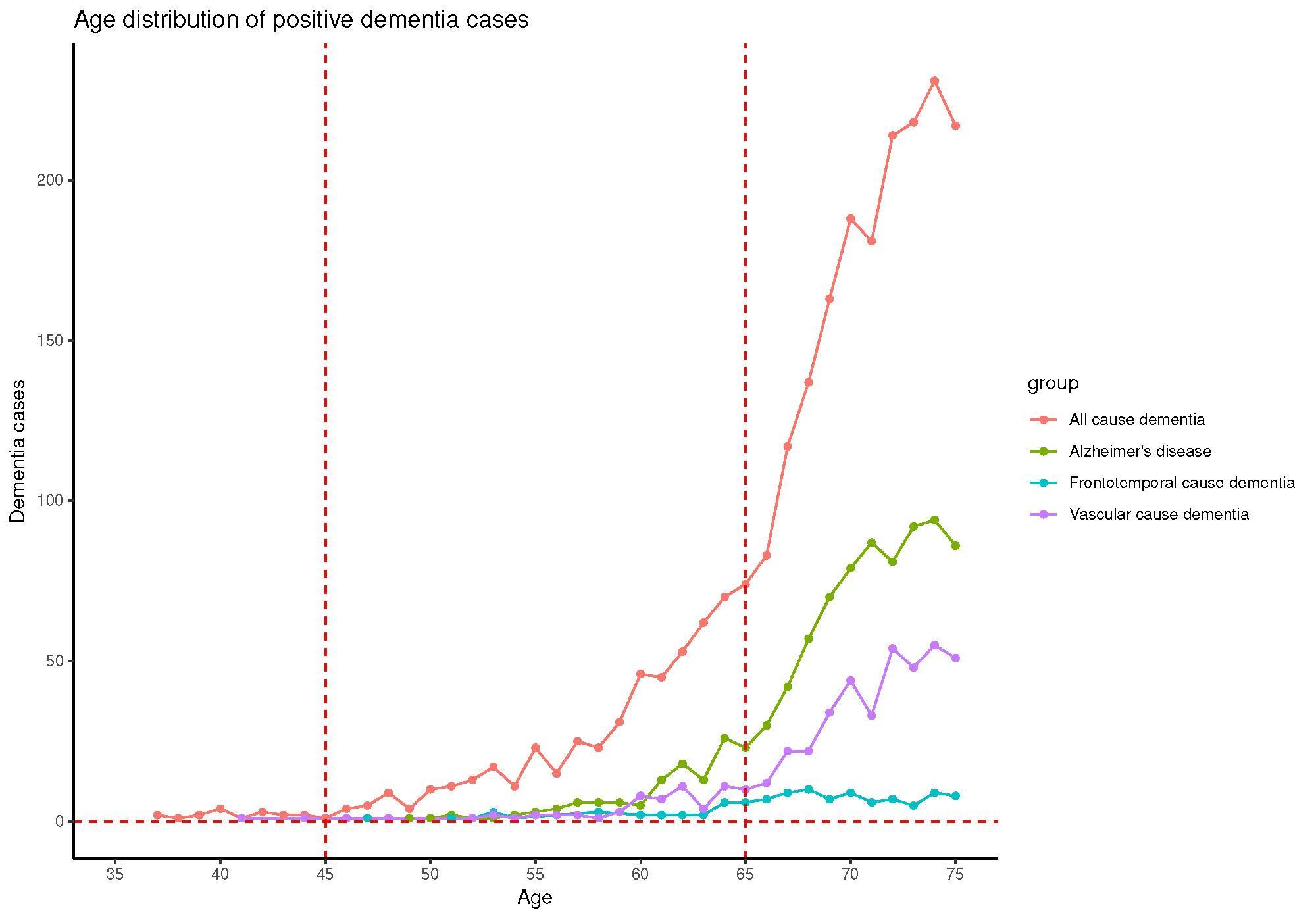


**Figure S1:** Distribution of participants’ age when they first reported their dementia status. The two vertical lines represent the two age cutoffs we used in this study, 45 and 65 years old.


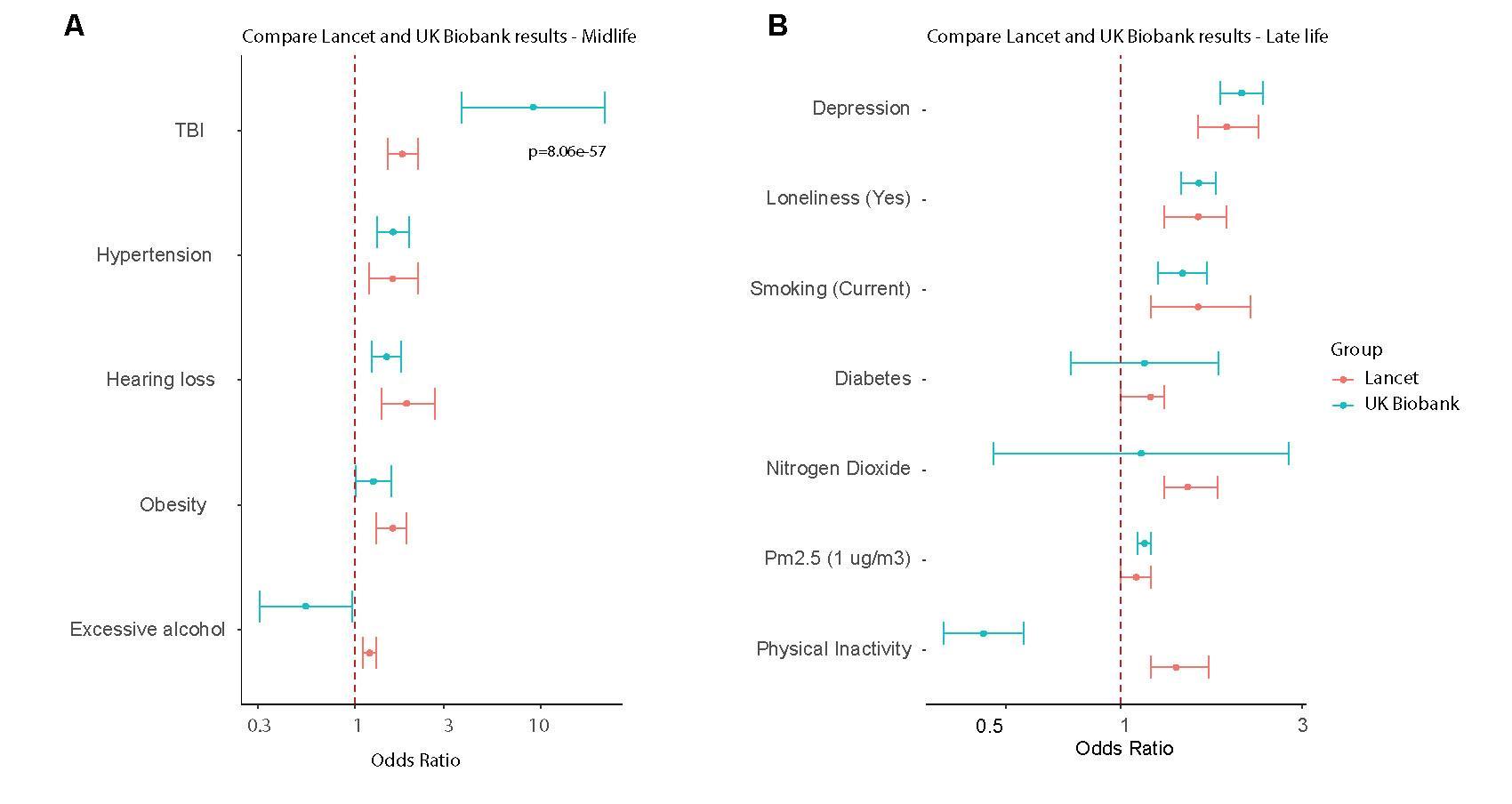


**Figure S2:** Comparison of the odds ratios and confidence intervals on the common risk factors from the Lancet 2020 report and the UK Biobank. We used the results from age and gender-adjusted models from the all-cause dementia participants from UK Biobank for comparison. The odds ratios from the gender-adjusted models of the midlife modifiable risk factors from the Lancet 2020 report compared to our UK Biobank results (**A**) and the late-life modifiable risk factors odds ratio comparison (**B**). Only significant p-values are shown in the figure.


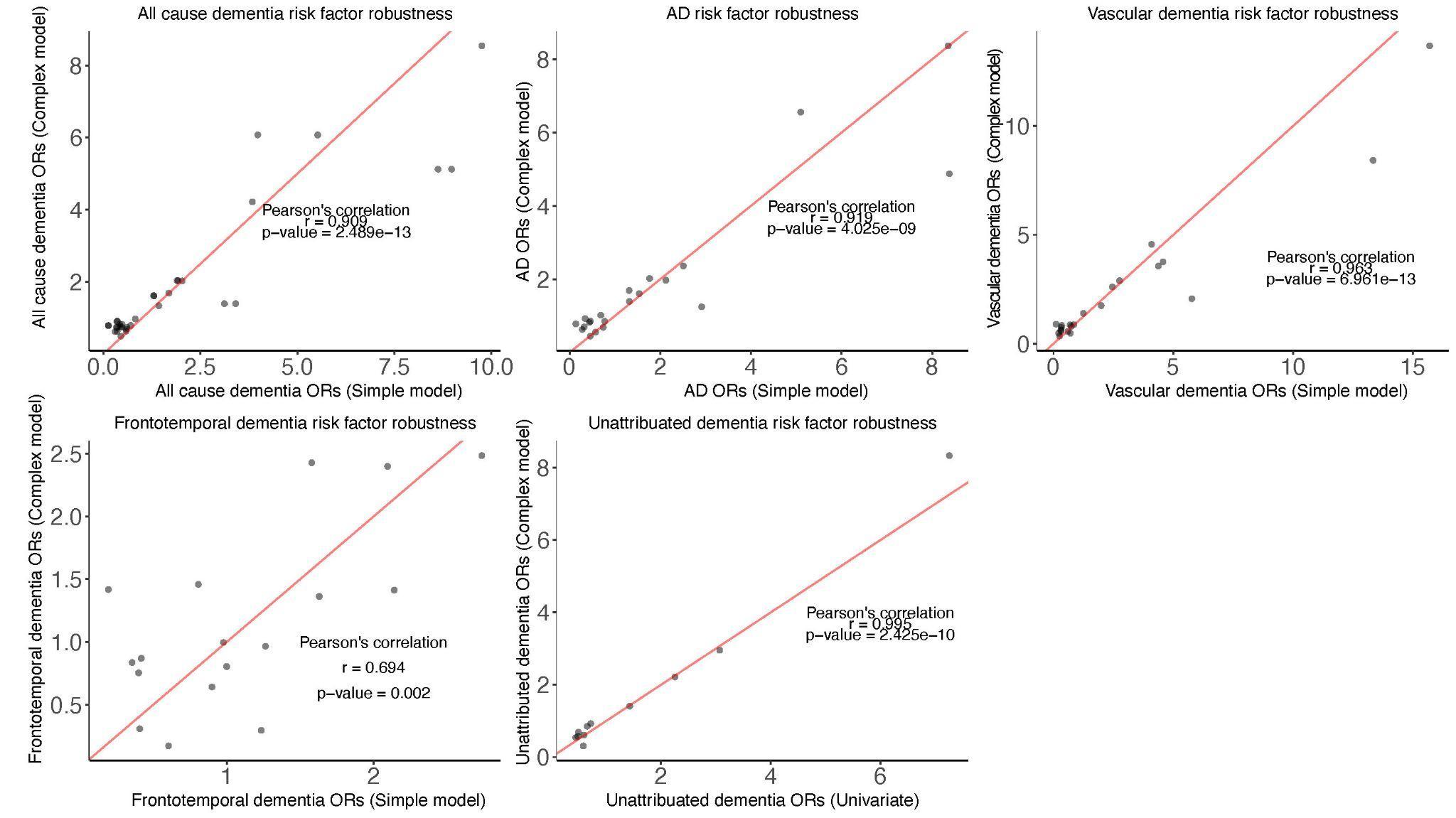


**Figure S3:** Correlation between the simple and complex models for dementia subtypes. The Pearson's correlations and p-values between the ORs from the no demographic adjusted models (simple) and multivariate models adjusting for age, gender, race, and all selected risk factors (complex) for all risk factors from both age groups. Each dot represents a risk factor. The red line indicates OR = 1.


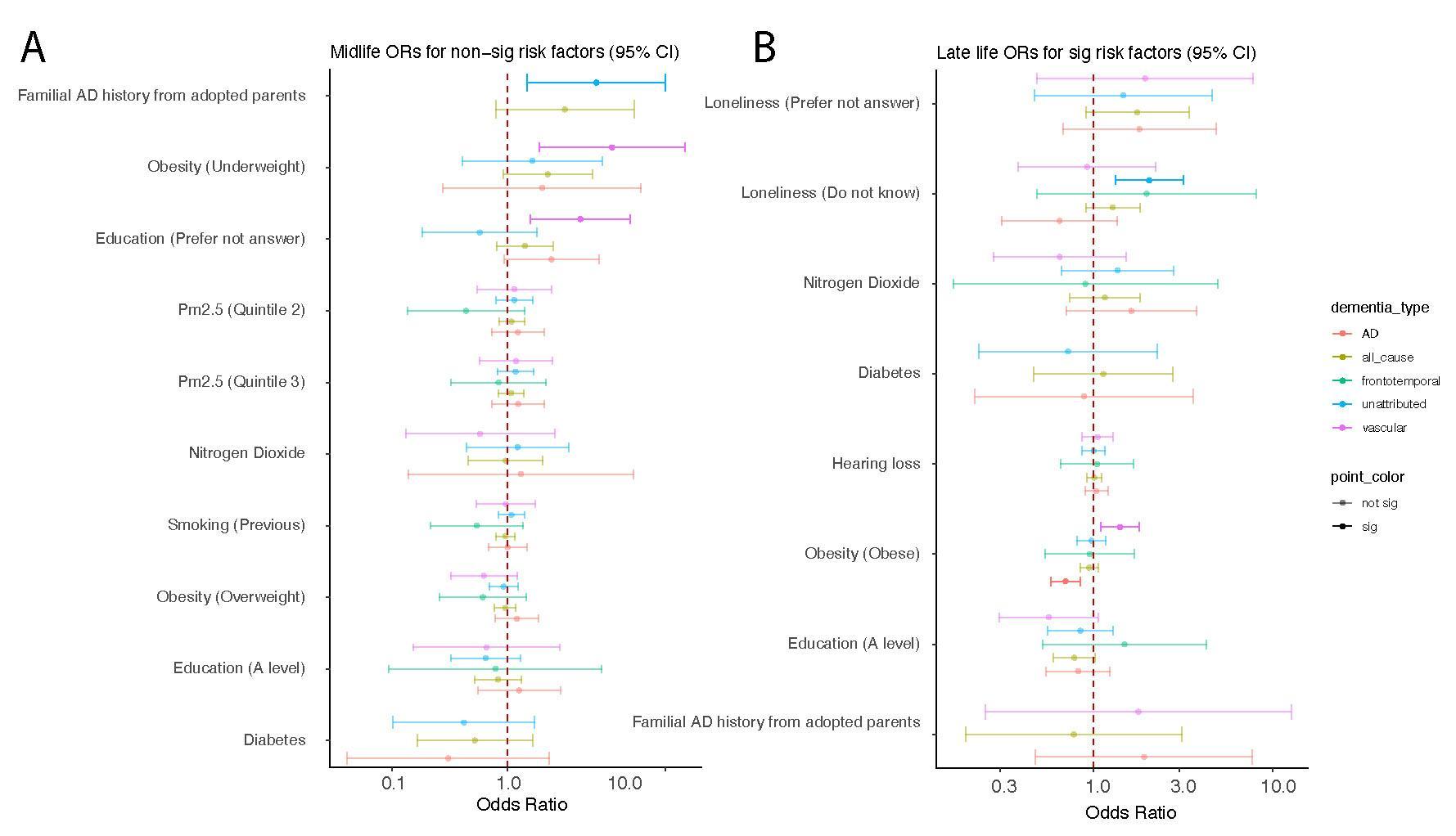


**Figure S4:** The ORs and 95% confidence intervals from gender- and age-adjusted models from non-significant all-cause dementia outputs ranked by the ORs from high to low in midlife (**A**) and late-life (**B**) groups. The ORs in the plots are colored by the dementia subtypes, and transparency indicates the significance.


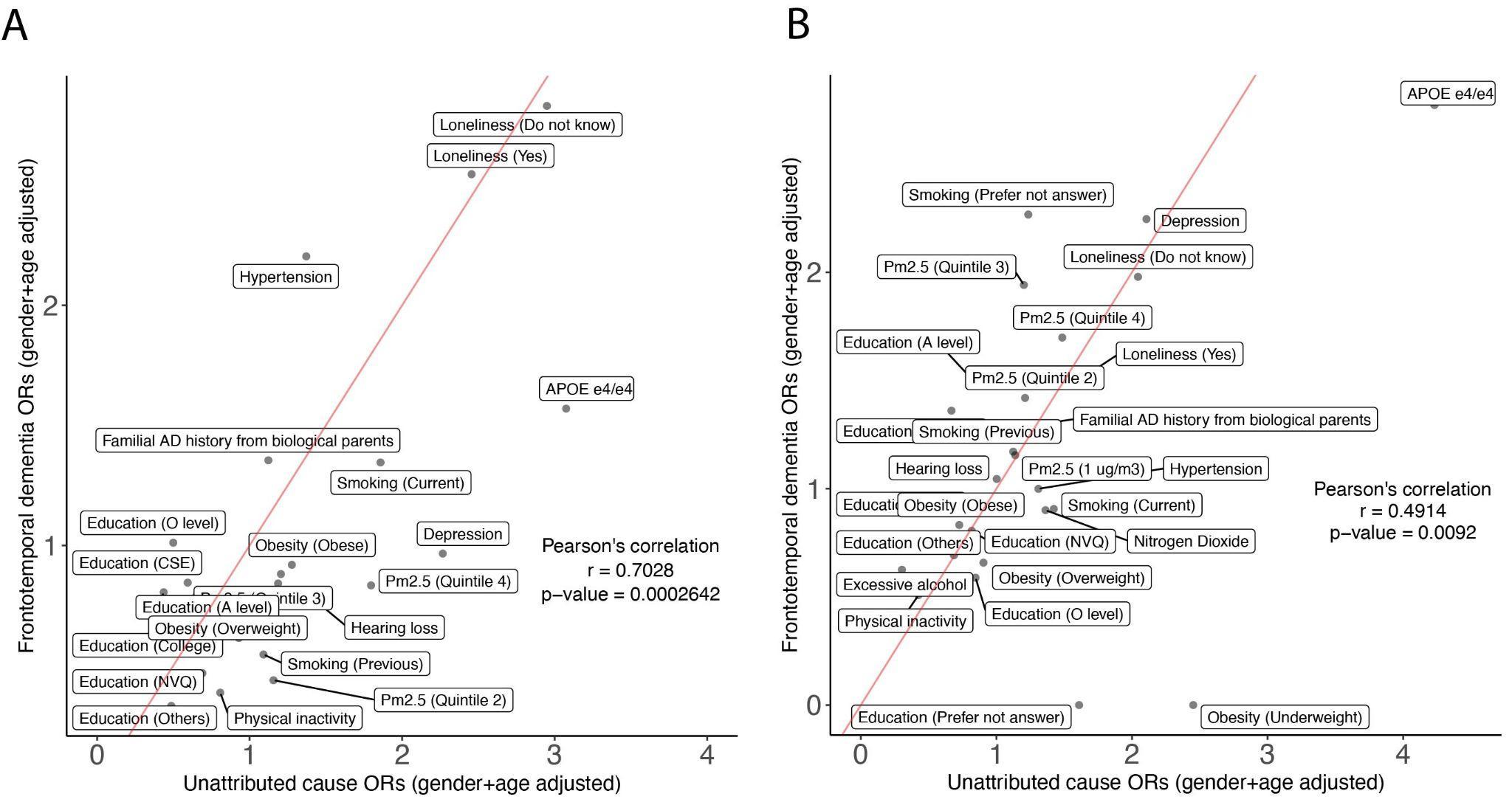


**Figure S5**: Correlation between frontotemporal dementia and the unattributed cause of dementia participants. The correlation plots above show the correlation in ORs on the risk factors between the unattributed cause dementia participants and the frontotemporal dementia participants. The Pearson correlations are reported, and the red line on the plots indicates when the slope is 1.
