# Supplementary material for: Specification curve analysis to identify heterogeneity in risk factors for dementia: findings from the UK Biobank
Source: BMC Med. 2024 May 29;22:216. doi: 10.1186/s12916-024-03424-w (PMC11134914; doi:10.1186/s12916-024-03424-w)
Supplement: Supplementary file 2 — Additional file 2: Supplementary Table S1. Definitions of risk factors. Supplementary Table S2. Prevalence of different dementia risk factors in different age groups. Supplementary Table S3. Summary stats for odds ratios from selected risk factors (Fig. 4). Supplementary Table S4. Gender and age adjusted model outputs in midlife and late life group. [file 12916_2024_3424_MOESM2_ESM.docx]

Additional File 2: Supplementary Tables

**
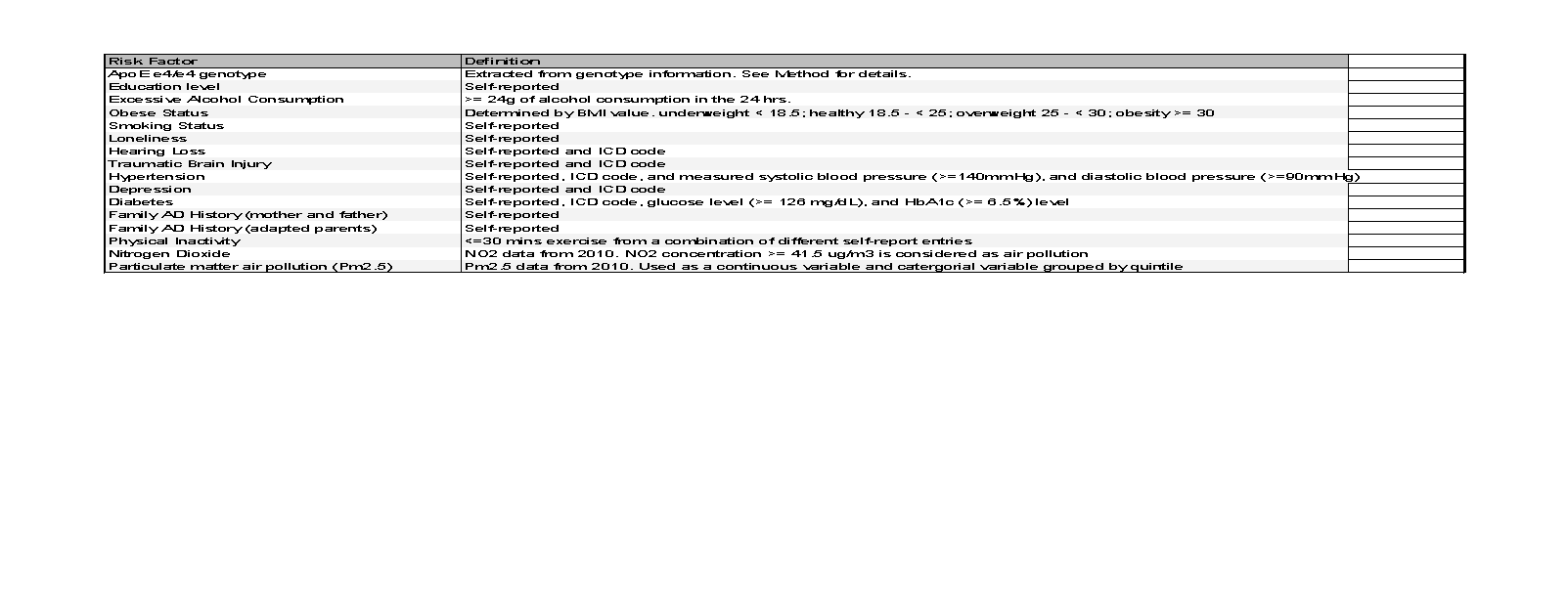
**

**Supplementary Table S1**: Definitions of risk factors

**
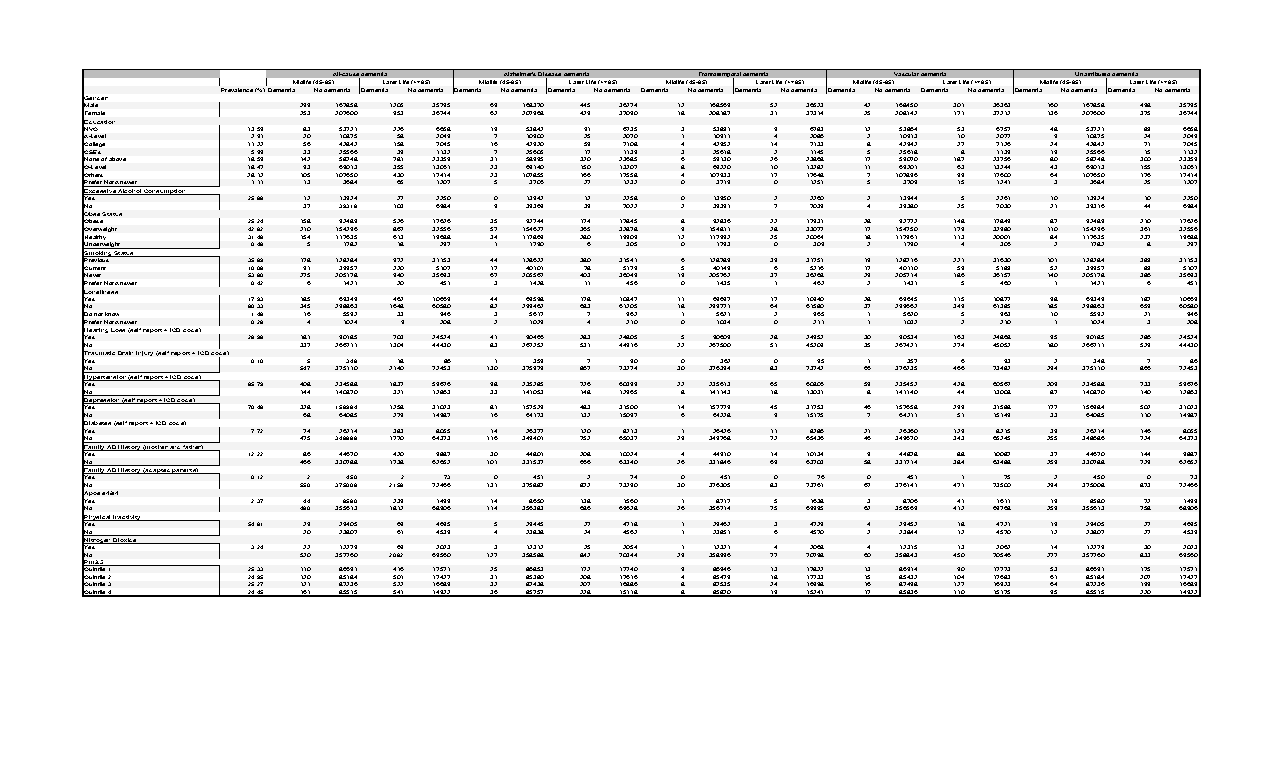
**

**Supplementary Table S2**: Prevalence of different dementia risk factors in different age groups

**
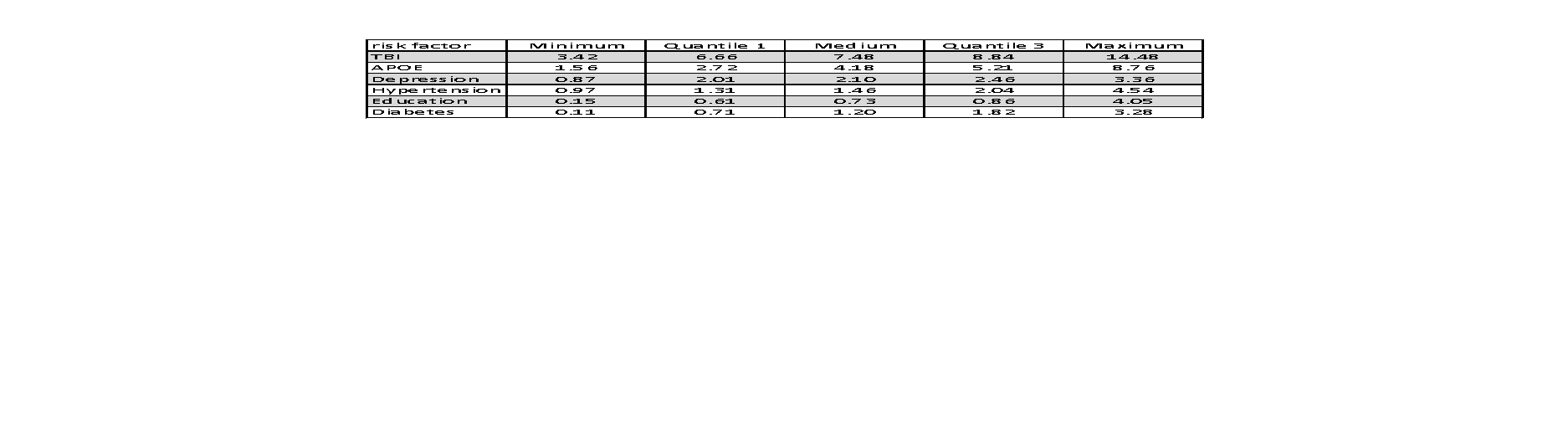
**

**Supplementary Table S3**: Summary stats for odds ratios from selected risk factors (Figure 4)

**
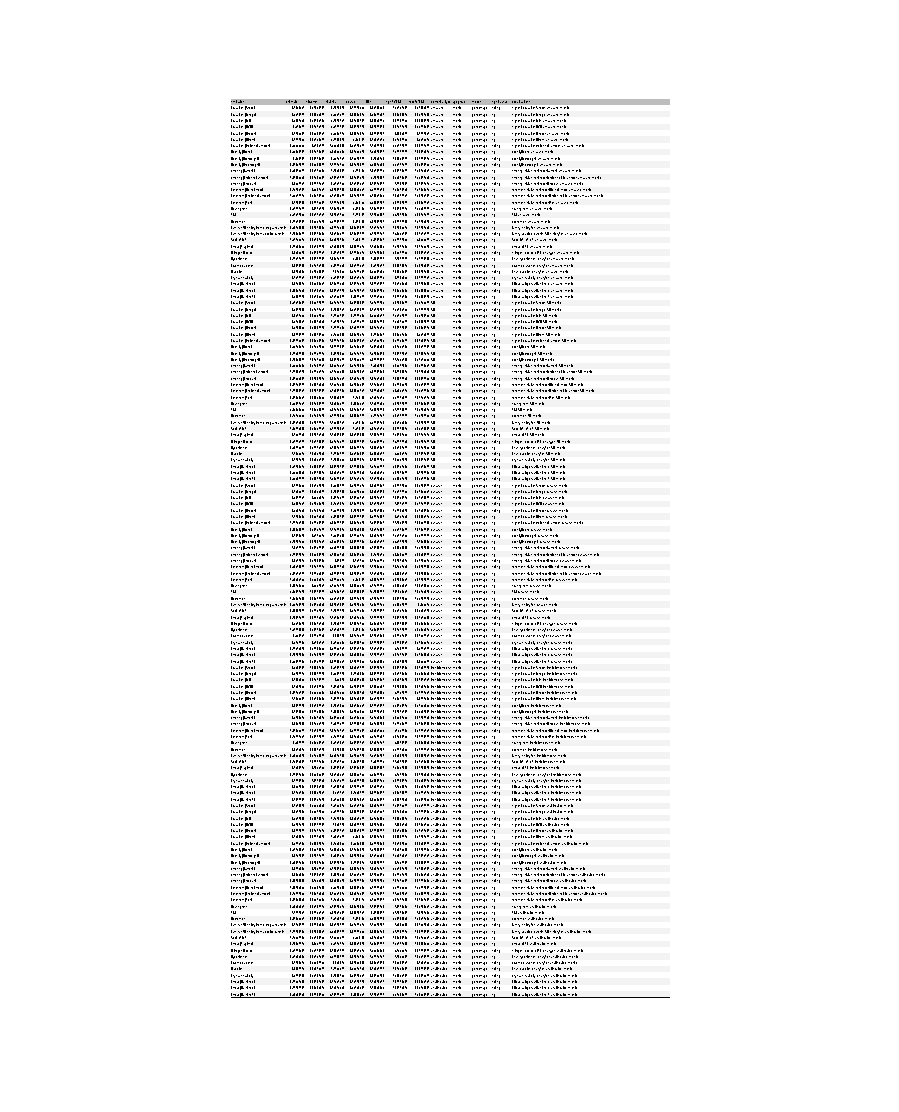
**

**
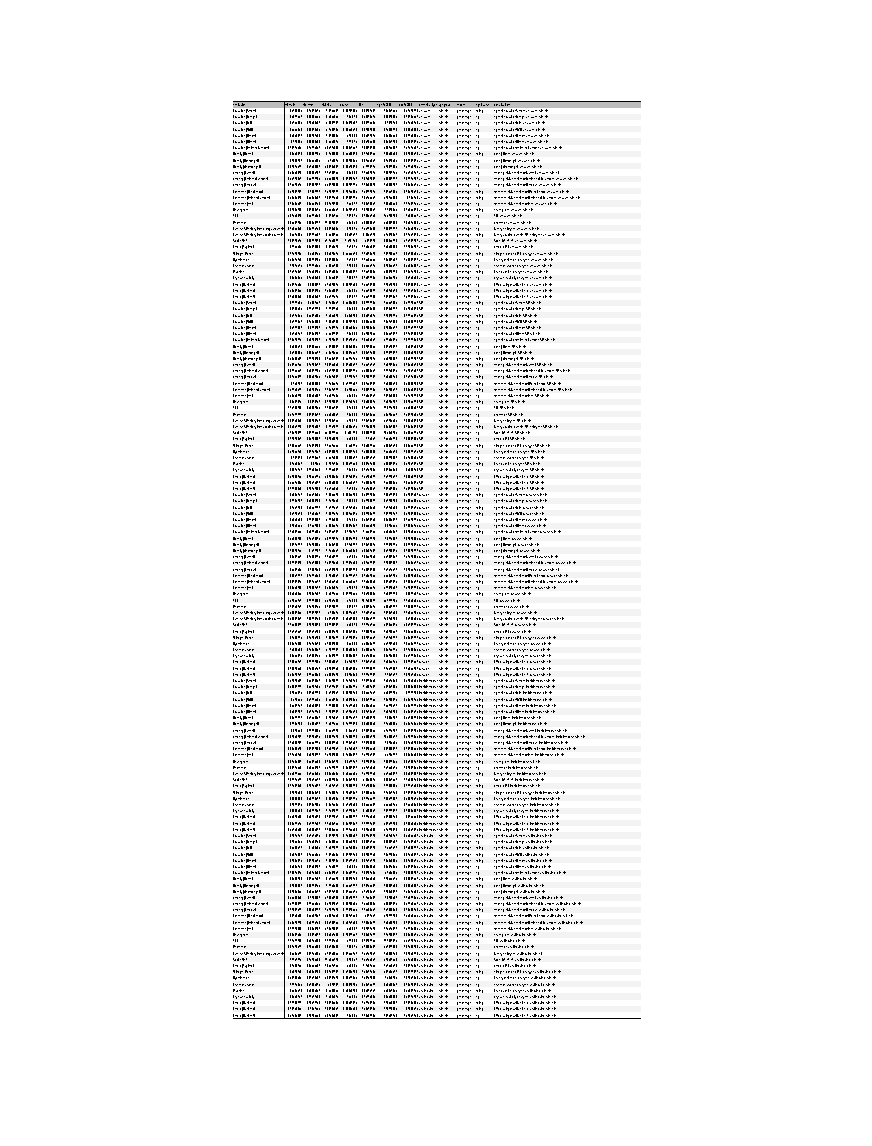
**

**Supplementary Table S4**: Gender and age-adjusted model outputs in midlife and late life group.
